# Conservation of a Chromosome 8 Inversion and Exon Mutations Confirm Common Gulonolactone Oxidase Gene Evolution Among Primates, Including *H. Neanderthalensis*

**DOI:** 10.1007/s00239-024-10165-0

**Published:** 2024-04-29

**Authors:** Alexander Mansueto, Deborah J. Good

**Affiliations:** 1 Department of Human Nutrition, Foods, and Exercise, Virginia Tech, Blacksburg, VA USA; 2 Department of Human Nutrition, Foods, and Exercise, Virginia Tech, 1981 Kraft Drive (0913), ILSB Room 1020, Blacksburg, VA 24060 USA; 3 Present Address: Department of Biological Sciences, Vanderbilt University, Nashvile, TN USA

**Keywords:** Ascorbic acid, Vitamin C, GULO, GULOP, Neanderthal, Primates, Phylogeny

## Abstract

Ascorbic acid functions as an antioxidant and facilitates other biochemical processes such as collagen triple helix formation, and iron uptake by cells. Animals which endogenously produce ascorbic acid have a functional gulonolactone oxidase gene (*GULO*); however, humans have a *GULO* pseudogene (*GULOP*) and depend on dietary ascorbic acid. In this study, the conservation of *GULOP* sequences in the primate haplorhini suborder were investigated and compared to the *GULO* sequences belonging to the primates strepsirrhini suborder. Phylogenetic analysis suggested that the conserved *GULOP* exons in the haplorhini primates experienced a high rate of mutations following the haplorhini/strepsirrhini divergence. This high mutation rate has decreased during the evolution of the haplorhini primates. Additionally, indels of the haplorhini *GULOP* sequences were conserved across the suborder. A separate analysis for *GULO* sequences and well-conserved *GULOP* sequences focusing on placental mammals identified an in-frame *GULO* sequence in the Brazilian guinea pig, and a potential *GULOP* sequence in the pika. Similar to haplorhini primates, the guinea pig and lagomorph species have experienced a high substitution rate when compared to the mammals used in this study. A shared synteny to examine the conservation of local genes near *GULO*/*GULOP* identified a conserved inversion around the *GULO*/*GULOP* locus between the haplorhini and strepsirrhini primates. Fischer’s exact test did not support an association between *GULOP* and the chromosomal inversion. Mauve alignment showed that the inversion of the length of the syntenic block that the *GULO*/*GULOP* genes belonged to was variable. However, there were frequent rearrangements around ~ 2 million base pairs adjacent to *GULOP* involving the *KIF13B* and *MSRA* genes. These data may suggest that genes acquiring deleterious mutations in the coding sequence may respond to these deleterious mutations with rapid substitution rates.

**Supplementary Information:**

The online version contains supplementary material available at 10.1007/s00239-024-10165-0.

## Introduction

**Fig. 1:**
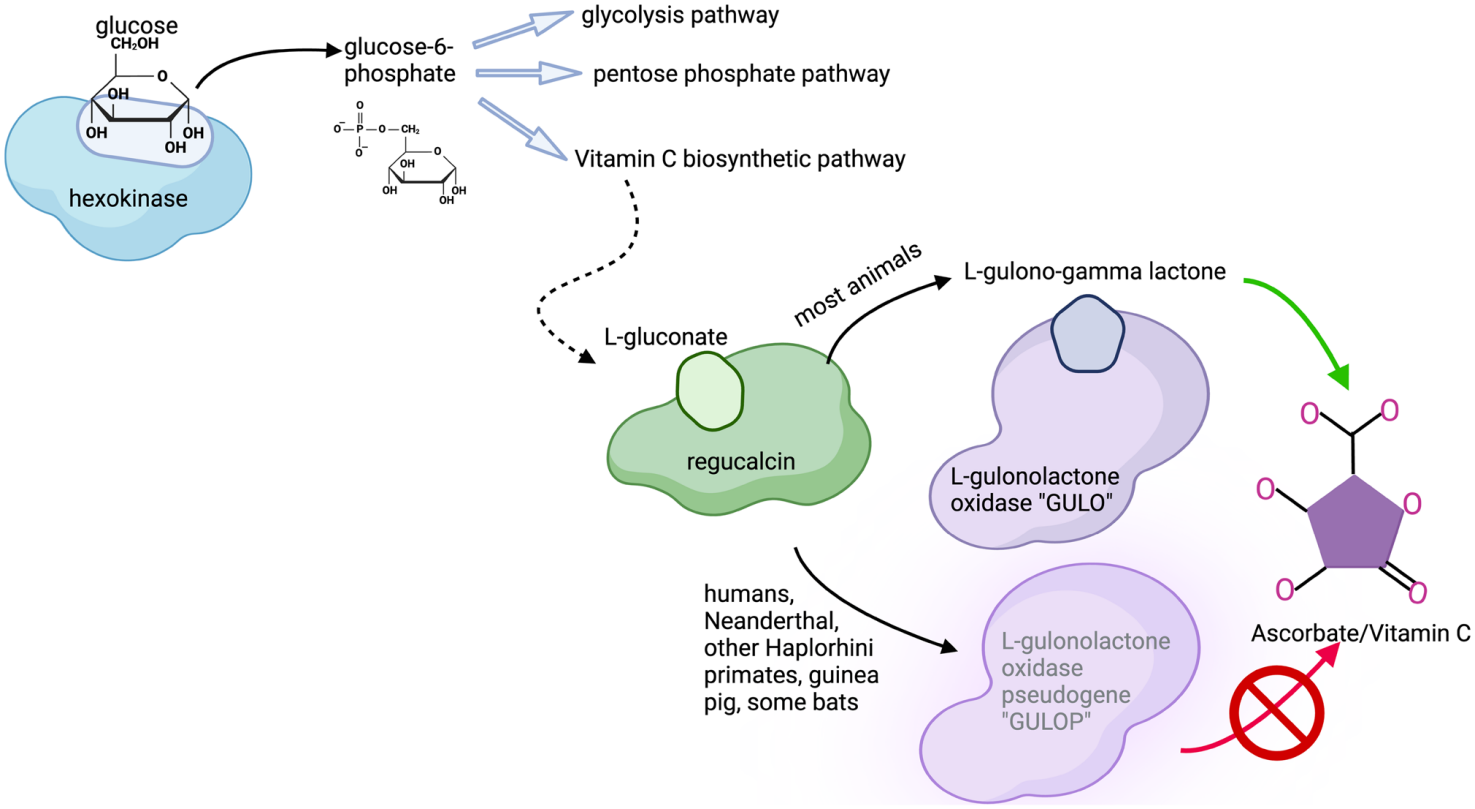
Ascorbic acid synthesis pathway in the presence of *GULO* or *GULOP.* Glucose is the starting carbohydrate for ascorbic acid synthesis. Phosphorylation of glucose by hexokinase forms glucose-6-phosphate which can be shuttled to the ascorbic acid synthesis pathway or used in glycolysis or the pentose phosphate pathway. In animals that have a functional *GULO* gene, the L-gulono-gamma lactone precursor is converted to ascorbate (vitamin C), but in animals with a *GULOP* gene, the pathway is non-functional. Those animals require dietary vitamin C and are prone to scurvy in its absence

The gulonolactone oxidase gene (*GULO*) encodes L-gulonolactone oxidase (Gulo), a key enzyme in animals that provides an alternative metabolic pathway for D-glucuronate degradation (Chatterjee et al. 1960; Nishikimi and Yagi 1991). In this alternative pathway, the enzyme regucalcin commits the intermediate molecule L-gulonate to L-gulono-1,4-lactone which can be converted to L-ascorbate (vitamin C) by the Gulo enzyme (Fig. 1).

Despite the importance of L-gulonolactone oxidase in ascorbic acid synthesis, some animals cannot endogenously synthesize ascorbic acid, and consequently these animals may develop scurvy if their diets lack significant sources of ascorbic acid (Chatterjee et al. 1960; Drouin et al. 2011; Linster and Van Schaftingen 2007; Nishikimi et al. 1992; Nishikimi and Yagi 1991). Dr. James Lind, in what was considered the first human clinical trial, demonstrated that sailors given a ration of oranges and lemons were cured of scurvy within about six days of eating them (Lind 1753). The lack of a Gulo enzyme was first documented by Grollman and Lehninger in 1957 (Grollman and Lehninger 1957) when they found that the liver in mammals and the kidney in birds and reptiles contains the vitamin C synthetic pathway. Specifically, they identified the three enzymes and vitamin C synthesis in rat, mouse, rabbit, dog, cat, pig, cow, chicken, pigeon, and turtle, but were unable to find Gulo in guinea pig, cynomolgus monkey, rhesus monkey and 5 different humans (Grollman and Lehninger 1957). Grollman and Lehninger noted that the four mammals which did not have the third enzyme were obligate consumers of vitamin C. Subsequent papers by Nishimiki and colleagues (Nishikimi et al. 1992, 1988; Nishikimi and Yagi 1991) identified an aberrant and non-expressing *GULO* gene in both humans and the domestic guinea pigs. This irregular *GULO* gene was identified as a unitary pseudogene of *GULO* (*GULOP*). Though some pseudogenes have functional roles (Petrov and Hartl 2000; Pink et al. 2011), *GULOP* does not have a known functional role. The bat genera *Pteropodidae, Noctilionida, Mormoopidae, Phyloostomidae, Natalidae, Vespertilionidae, and Molossidase* have lost Gulo enzymatic activity (Cui et al. 2011a) by a stepwise accumulation of mutations during *GULO* evolution in these species (Cui et al. 2011b). Interestingly in bats, the more recent the speciation event for the bat, the more accumulated mutations were identified, with Cui and colleagues hypothesizing that the *GULO* gene was becoming redundant in bats (Cui et al. 2011b). A diagram of the ascorbic acid synthesis pathway is shown in Fig. 1.

There are two primate suborders which are the haplorhini and strepsirrhini primates. These suborders are estimated to have diverged 70 million-years-ago (MYA) (Pozzi et al. 2014). *GULOP* is found in all haplorhini primates while the strepsirrhini primates have a functional *GULO* gene (Drouin et al. 2011). Within the haplorhini suborder are the hominid primates consisting of the genera *Pongo*, *Gorilla*, *Pan*, and *Homo* which are predicted to have diverged 12–16 MYA (Pozzi et al. 2014). Though pseudogenes like *GULOP* have equal synonymous and nonsynonymous mutation rates (Lachapelle and Drouin 2011; Nishikimi and Yagi 1991), this relatively short divergence time among the hominids suggests that there may be high *GULOP* sequence similarity between the genera.

Potential Neanderthal bones date from 350,000 years ago, but most fossils are from 130,000 years and earlier, with the transition to modern humans ~ 40,000 years ago (Villa and Roebroeks 2014). Thus, like the bat story, evolution of Hominids (which include extinct and modern Great Apes) occurs in the more recent evolutionary history. The Neanderthal genome was not available when the *GULOP* pseudogene was originally described. Thus, the question arises as to whether, like bats recent hominid evolution show a similar accumulation of mutations, compared to more ancient members of the *Hominidae* family. The Neanderthal draft genome was established from DNA found in the Vindija Cave located in Croatia, but as a draft genome, it did not have complete coverage of all regions, including those encompassing the chromosome 8 *GULO*/*GULOP* region (Green et al. 2010). In December 2013, the complete Neanderthal genome was obtained from the toe bone of a woman from Siberia (Prufer et al. 2014). This genome has 99.9% coverage of at least tenfold, and an average of 50-fold for all unique regions of the genome (https://bioinf.eva.mpg.de/) (2015).

In this study, the *GULO* or *GULOP* genes from haplorhini and strepsirrhini primates was studied with phylogenetic analysis to examine the evolution of the remaining homologous exons to test the hypothesis that more recent speciation events in *Hominidae* would accumulate more variants in the *GULO* gene. Particular attention in this analysis was given to the Neanderthal *GULOP* sequence because this species most recently diverged from humans between 680,000 and 350,000 years ago (Pozzi et al. 2014; Villa and Roebroeks 2014). The *GULOP* sequence of the haplorhini primates has significantly degenerated from the common 12 exon and 440 amino acid long sequence. Therefore, a separate phylogenetic analysis was conducted on mammals with well-conserved *GULOP* sequences for a robust comparison of mutations between *GULO* and *GULOP* sequences. Lastly, the *GULO* and *GULOP* sequences are positioned on opposite strands of the haplorhini and strepsirrhini primates. We hypothesized that the strand position of *GULO* may be associated with the occurrence of its pseudogene among mammals. Chromosomal inversions around *GULO* were scored and a Fischer’s exact test was conducted which did not support our hypothesis. Despite this finding, the orientation of the syntenic *CLU* gene is consistently anti-parallel to *GULO*/*GULOP*, and it may be a useful landmark to identify the *GULO*/*GULOP* gene in mammalian genomes where it is not annotated.

## Material and Methods

### Sequence Acquisition

*GULO* and the human *GULOP* sequences were acquired from Ensembl™ Release 107 (Zerbino et al. 2018) or from NCBI (Supplemental File 1). The Altai Neanderthal chromosome 8 sequence was acquired from the Max Plank Institute for Evolutionary Anthropology (Green et al. 2010; Prufer et al. 2014). These data are made freely available for individuals that are studying an individual gene or individual features of the genome. The Integrative Genomics Viewer (IGV; https://software.broadinstitute.org/software/igv/) was used to align the human chromosome 8 sequence with the Neanderthal chromosome 8 sequence (Robinson et al. 2017). SNVs in the Neanderthal *GULOP* were identified by a 90% deviance allele threshold from the reference sequence.

*GULOP* sequences were identified by BLAT analysis with a *GULO* gene or *GULOP* sequence from a taxonomically similar species. BLAT differs from BLAST in that BLAT allows for faster analysis by building an index of the database as opposed to indexing the query sequence, and BLAT can account for exon/intron splice sites in RNA and DNA alignments (Kent 2002). If nucleotides were missing on either the 5’ or 3’ ends of the BLAT hit when compared to *GULO* exon sequences, an additional set of nucleotides corresponding to the missing nucleotides were added after BLAT analysis. All BLAT hits (edited or unedited) were then reciprocally BLAT against the former query sequence (Supplemental File 1). Only the nucleotide sequences aligned to the reciprocal BLAT were accepted as *GULOP* sequences. For example, a BLAT using human *GULOP* as the query against the Gorilla genome was used. A preliminary Gorilla *GULOP* sequence was acquired. Then, the preliminary Gorilla *GULOP* sequence was BLAT back to the human genome. Only the reciprocating BLAT sequence from this search was used in all analyses.

### Phylogenetic Analyses

The MEGA XI: Molecular Evolutionary Genetics Analysis across computing platforms (https://www.megasoftware.net/) (Tamura et al. 2021) program was used for the generation of the multiple sequence alignments (MSA) and phylogenetic trees. The Clustal Omega algorithm with default settings was used to generate the MSA. All sites where gaps occurred were removed from the analysis. Substitution models were simulated in MEGA XI with a maximum composite likelihood statistical method and using the Tamura-Nei model. The substitution model with the lowest corrected Akaike Information Criterion (AICc) value was used for rendering Bayesian inferred phylogenies using MrBayes. MrBayes was used for phylogenetic tree rendering, and the number of iterations used was dependent upon when the average standard deviation in an analysis was below 0.01. MrBayes uses Metropolis coupling which allows for parallel sampling of phylogenetic trees using heated and cold chains (Altekar et al. 2004). The parameters Swapfreq, Nswaps, Nchains, and Temp establish the number and behavior of the heated and cold chains. These parameters were left at the default values provided by MrBayes which specifies for 3 heated chains and 1 cold chain. Additionally, one chain was swapped upon every MCMC iteration. Consensus trees were generated with a 25% burn-in, and the consensus trees were subsequently rooted in FigTree version 1.4.4.

### Pairwise Distance Analysis

The pairwise distances are calculated as nucleotide substitutions per site. The Tamura 3-parameter model with a gamma rate among sites of 1 was used to calculate the pairwise distances between species. All sites where gaps were present were deleted from the analysis. Both transition and transversion substitutions were included. This analysis was conducted in MEGA XI (Tamura and Nei 1993; Tamura et al. 2021).

### Exon/Intron Analysis

The exon/intron analyses were conducted with NNSPLICE 0.9. Briefly, the coding strand of a *GULO* or *GULOP* sequence of interest was submitted to NNSPLICE 0.9 which was operated with default settings.

### Shared Synteny

Bat species not included in the phylogeny were included for the synteny to increase the representation of *GULOP* sequences in forward and reverse directions. Species represented in the gene synteny analysis are included in Supplemental File 1. All species were independently examined for the genetic rearrangement events which may have occurred around *GULO*/*GULOP* and between the genes *STMN4* and *NUGGC* or *STMN4* and *BAG1*. Species with identical orders, families, or genera and identical chromosome arrangements were excluded from this analysis to prevent redundant counting. Small gene fragments and non-coding genes which occurred between the synteny range were disregarded. For example, the haplorhini primates have identical genomic arrangements around *GULOP* with some non-coding genes interspersed between the synteny range. Therefore, only the human chromosome 8 region was included to represent all haplorhini primates. A Fischer’s exact test was conducted with R 4.0.3.

### Mauve Alignments

The chromosome and scaffold assemblies where *GULO* or *GULOP* is located was downloaded as a full Genbank sequence from NCBI for human, grey mouse lemur, dog (reference genome), cat, mouse (C57Bl/6 J), domestic guinea pig, pig (reference genome), and sheep. A progressive Mauve alignment was used for whole chromosome alignment between species of the same order (Darling et al. 2010). The seed size is automatically determined by the algorithm based on genome size. This seed size is multiplied by a value of three to determine locally colinear blocks (LCBs). The LCBs are regions of similar nucleotide identity which encode orthologous genes despite genomic rearrangements along a chromosome. Using the LCB weight slider in the Mauve GUI, a new LCB weight value was determined for each alignment performed, and the analysis was repeated with the new LCB weight value to remove spuriously identified LCBs. These final alignments were used for analysis.

## Results

### Analysis of Primate GULO Sequences

**Fig. 2:**
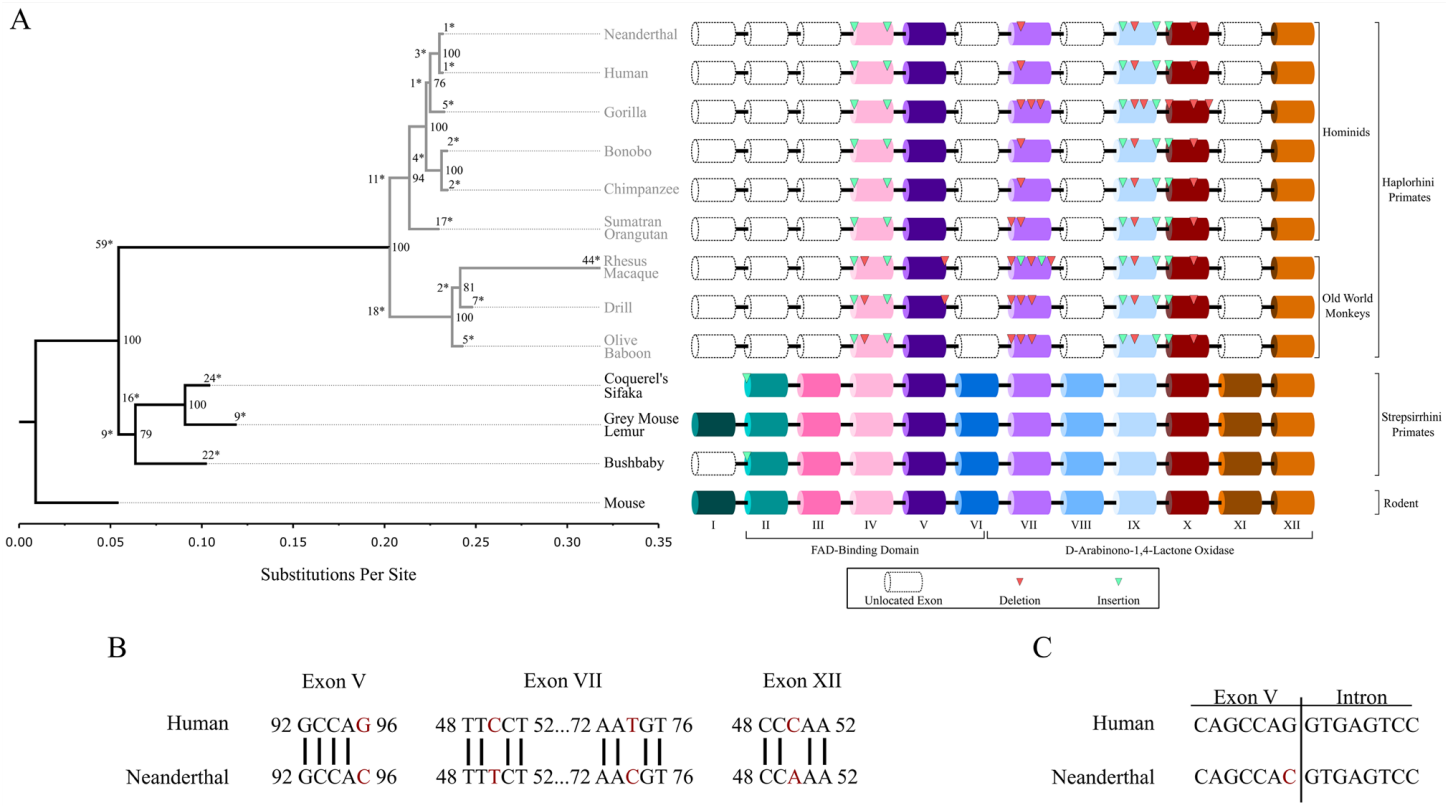
*GULO* and *GULOP* gene structure among primates: **A** Phylogenetic tree showing *GULO* evolution among the primates. *GULO*/*GULOP* sequences were aligned in MEGA 11 with the Clustal Omega tool. Substitution matrices were calculated in MEGA11 with a complete deletion option for gaps and ambiguous sites. All sequences submitted for phylogenetic analysis had all gaps and ambiguous sites deleted. Bayesian-inferred phylogenies were rendered with 250,000 iterations in MrBayes with a GTR+G substitution model. The GTR+G model was the model with the lowest AICc value. A burn-in of 25% from the cold-chain generated phylogenies was used to generate a consensus tree. The tree was rooted to *Mus musculus* in FigTree version 1.4.4. A scale bar representing nucleotide substitution per site is provided. Numbers distinguished with an asterisk represent conserved substitutions at a node or unique substitutions of the species from the final alignment. Numbers without asterisks are the posterior probabilities of a node which were provided by MrBayes. An exon map showing the homologous exons of *GULO* and *GULOP* is provided next to each species. Exons in white with dashed lines are not detected. Indels are shown with light and dark triangles indicating insertions or deletions, respectively. **B** Alignment of *GULOP* exon sequences where SNVs occur between *Homo sapiens* and *Homo neanderthalensis* from untrimmed alignments. SNVs are labelled relative to their position in the exon sequence, and SNVs are indicated by the absence of a vertical line. **C** Exon/intron border of orthologous exon V and its 3’ intron. Exon/intron analysis was performed in NNSPLICE 0.9. Scores of 1.00 and 0.99 were computed for *Homo sapiens* and *Homo neanderthalensis*, respectively

The mammalian *GULO* gene commonly has 12 exons which encode a 440 amino acid long protein. The first exon in mammalian *GULO* contains a 5’ untranslated region and the ATG start codon. The subsequent exons proceeding exon I code for the FAD-binding domain and the D-arabinono-1,4-lactone binding domain of L-gulonolactone oxidase. haplorhini *GULOP* sequences retain identity to exons IV, V, VII, IX, X, and XII suggesting that all other exons have degenerated beyond recognition (Fig. 2A). Phylogenetic analysis was conducted using the general time reversible (GTR) model with gamma parameter of the homologous *GULO* exons. The phylogeny rendered follows the current speciation events of the primate lineage. A discrepancy in the depiction of speciation events by phylogeny is the placement of the Gorilla *GULOP* adjacent to the genus *Homo*. The phylogeny depicts a greater number of substitutions per site between the haplorhini and strepsirhini primates. Indeed, there are a total of 59 conserved substitutions which occur at the node of the haplorhini suborder in the phylogeny. Within the haplorhini suborder, there is a reduction in the number of substitutions per site indicated by the phylogeny when compared to the initial haplorhini and strepsirrhini split (Fig. 2A, Supplemental File 2). Examination of the individual substitutions between the old-world monkeys and the hominids show 18 and 11 unique and conserved substitutions. These substitutions are further reduced apart from the rhesus macaque which had 44 unique substitutions when compared with all other primates. Like the substitutions, a separate examination of the indels between *GULOP* sequences showed conservation of some indels within the haplorhini *GULOP* suborder (Supplemental Fig. 1, Supplemental File 2). These findings suggest that the mutations accumulated in the *GULOP* exons occurred primarily at the haplorhini and strepsirrhini split.

Since the human and Neanderthal species diverged 400,000 years ago, we were curious with the extent of drift that occurred at the *GULOP* locus. Four SNVs were identified between humans and Neanderthals (Fig. 2B). The Neanderthal SNV G96C at exon V occurs at the last nucleotide position of this exon boundary. Nucleotide substitutions at exon/intron boundaries may affect how exons and introns are identified. We tested whether the G96C SNV in exon V would affect the exon/intron boundary with NNSPLICE version 0.9 (Fig. 2C). The G96C SNV does not significantly affect the recognition of the Neanderthal exon V boundary with its 3’ intron.

### Evolution of the Mammalian GULO and GULOP Sequences

**Fig. 3:**
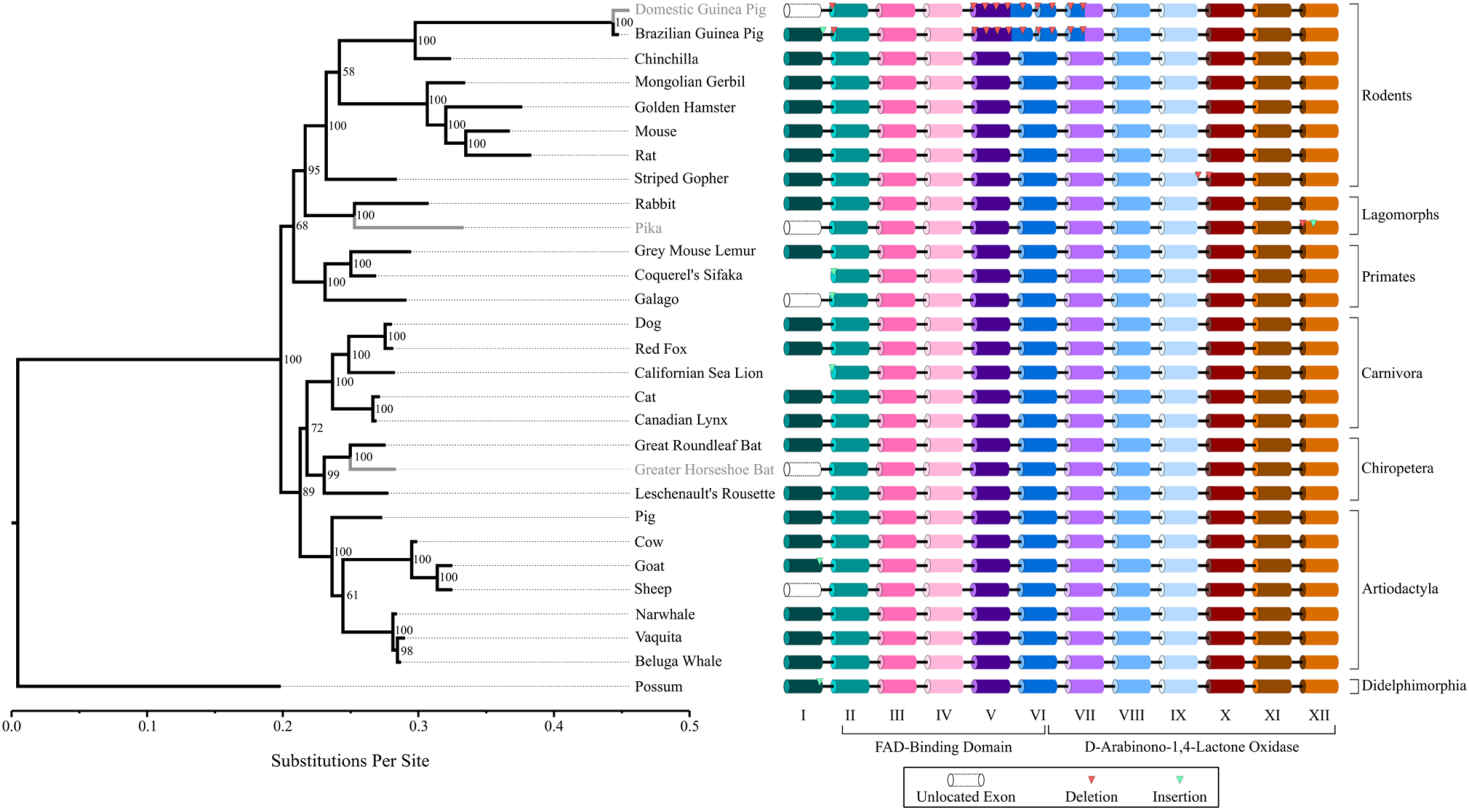
*GULO* and *GULOP* gene structure among mammals: DNA sequences of *GULO* and *GULOP* genes were acquired from Ensembl and NCBI databases. Sequences were aligned in MEGA11 with the Clustal Omega algorithm. A substitution matrix was calculated in MEGA11 with a complete deletion of gaps and ambiguous sites in the alignment. Trimmed sequences were used to generate Bayesian-inferred phylogenies with MrBayes. A GTR+G substitution model was used for modeling which had the lowest AICc value. 1,000,000 iterations were performed, and a consensus tree was drawn after discarding 25% of phylogenies from the cold chain. The phylogeny was rooted to the marsupial, possum, in FigTree 1.4.4. Exon/intron boundaries were tested with NNSPLICE 0.9. Taxa with phylogeny lines in grey contain either a pseudogene or do not express *GULO*. An exon schematic is provided for all species within the phylogeny where exons in white with dashed borders indicate exons which are not identified in the genome. Indels are shown by light and dark triangles indicating insertions and deletions, respectively

Next, we wanted to examine the evolution of other *GULO* and *GULOP* sequences with greater conservation of sequence information. A phylogeny of *GULO* was inferred with a GTR+G substitution matrix, which included placental mammals and the marsupial, possum, as an outgroup (Fig. 3). The haplorhini primates were not included in this analysis because a significant loss of nucleotide sequence would be incurred thus reducing the accuracy of the analysis. Phylogenetic analysis separates the species accordingly by order. In the placental mammal orders, the sequences have minor fluctuations in substitutions per site as indicated by the phylogeny, but the rodent order experiences a larger range in substitutions per site, especially in the families Muridae and Caviidae (Fig. 3, Supplemental File 2). Within the rodent order, the striped gopher *GULO* sequence is annotated by Ensembl™ as having two indels (Supplemental Fig. 2). NNSPLICE results suggest that there is a functional donor splice site and the 5-bp indel is an annotation error (Supplemental Fig. 3B). Additionally, the 1-bp deletion at the start of exon X in the striped gopher may be an annotation error as well, but NNSPLICE cannot identify the acceptor splice site for this exon in all species tested. Although the striped gopher is missing 6-bp from its *GULO* sequence, we used the Ensembl™ annotation for this analysis.

The domestic guinea pig has a well-described *GULOP* sequence, and we expected the Brazilian guinea pig to have a pseudogene as well. However, the Ensembl™ database has annotated a *GULO*-like gene in the genome of the Brazilian guinea pig. The main difference between the sequence of the domestic and Brazilian guinea pigs is an indel with either 2 or 1 nucleotides deleted, respectively (Supplemental Fig. 2). This 1 nucleotide deletion in the Brazilian guinea pig *GULO* maintains an open reading frame resulting in a putative 412 amino acid long sequence. Despite this gene being in-frame, there are significant rearrangements between exon V, exon VI, and exon VII along with numerous indels (Fig. 3, Supplemental Fig. 2). NNSPLICE version 0.9 was used to test if the resulting mRNA transcript could be properly spliced. The intron acceptor sites of these rearranged exons seem intact relative to the mouse *GULO* gene (Supplemental Fig. 3C). However, the exon donor splice sites in exons II, V, and VI were not identified (Supplemental Fig. 3D). Additionally, there is a high substitution rate identified in the *Cavia* genus which is similar to what was identified in the haplorhini primates.

Within the lagomorph order, the rabbit has an annotated *GULO* gene in Ensembl™, but the *GULO* gene in pika was not annotated by Ensembl™. Using the BLAT analysis, *GULO*-like exons were identified in the pika genome. We identified a 4-bp deletion and a 1-bp insertion in exon XII (Fig. 3, Supplemental Fig. 2). NNSPLICE version 0.9 was used to check if the pika exon XII acceptor site was functional (Supplemental Fig. 3A). NNSPLICE analysis suggests that the final 4-bp of the intron are duplicated and replace the 4-bp deletion identified by reciprocal BLAT. This splice site is moderately supported at the beginning of exon XII in pika. Although this 4-bp sequence was not included in the phylogenetic analysis, its inclusion would result in a frameshift mutation and the skipping of the termination codon during translation of this *GULO* transcript. Alternatively, the exon XII acceptor splice site of the pika *GULO* may not be recognized which would prevent the proper splicing of the mRNA transcript. Therefore, these results suggest that the *GULO* gene of pika is nonfunctional.

Similar to haplorhini primates, the chiroptera order was originally identified as having a pseudogene for *GULO* (Birney et al. 1976). However, previous work suggest that some bat species have spontaneously regained functionality of *GULO* (Drouin et al. 2011). The two bat species represented on the phylogeny have a similar substitution per site rate relative to other mammals (Fig. 3). This suggests that the *GULO* and *GULOP* sequences of the bats represented here may be a recent evolutionary event and has not diverged significantly from *GULO* despite transient reactivation or complete pseudogenization of the gene.

### Gene Synteny Analysis of Chromosomal Inversion Around GULO

**Fig. 4:**
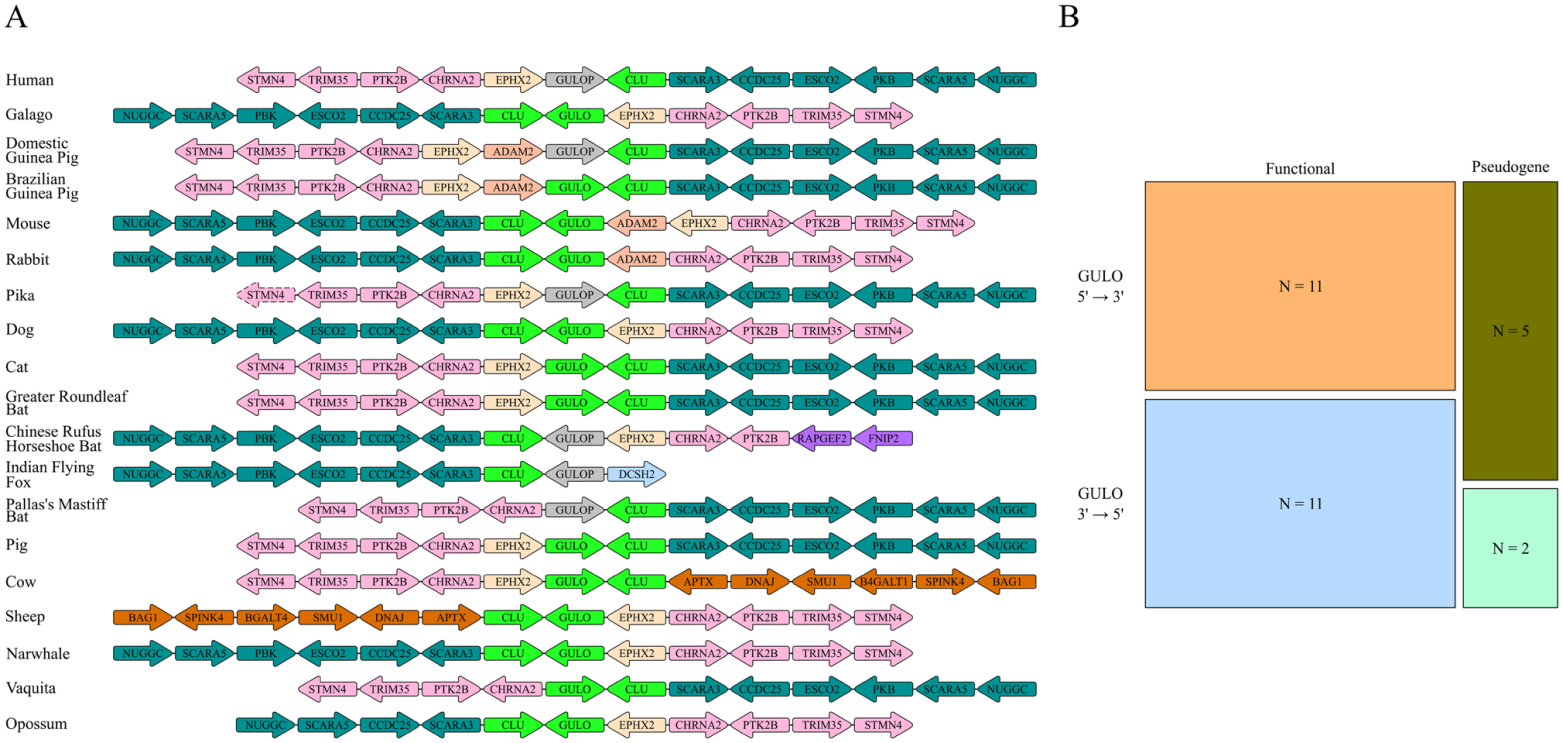
Synteny Analysis of Vertebrate *GULO*/*GULOP* Chromosome Regions: Species chromosomes were analyzed for independent chromosome rearrangements. Species order, family, and genera were considered with respect to *GULO* functionality and orientation when deciding on which species to include for analysis. **A** Synteny with representative species showing the genetic composition from the *NUGGC* gene to the *STMN4 *gene. The synteny is centered around *GULO/GULOP*. Coloring indicates syntenic blocks (*GULOP* is colored grey to distinguish it from *GULO*). **B** A Fischer’s Exact test was performed to test the hypothesis that the *GULO* functionality is dependent on the *GULO* orientation. This test was performed with R, and a p-value of 0.41 was calculated

When searching for *GULOP* sequences in haplorhini primates, we used the syntenic *CLU* gene to identify where *GULOP* occurred in the primate genomes (Yang 2013). We noticed that the haplorhini primates examined had a chromosomal inversion of the surrounding genes relative to the strepsirrhini primates (Fig. 4A; Human and Galago). This chromosomal inversion suggests that the primate chromosome containing *GULO* (chromosome 8 in humans) was inverted at the haplorhini and strepsirrhini suborder split. Further examination of the chromosome region between *STMN4* and *NUGGC* showed similar chromosomal rearrangement events occurring between the Caviidae and Muridae families and the rabbit and pika species. In the chiroptera order, there are few chromosomal assemblies for these animals, and the greater horseshoe bat previously analyzed does not have an assembly at this time. Therefore, we expanded the number of bat genomes for the synteny analysis and observed frequent chromosomal inversions around the *GULO*/*GULOP* locus (Fig. 4A).

The syntenic blocks frequently identified are *STMN4* to *CHRNA2*, *EPHX2*, and *SCARA3* to *NUGGC* which occur around the *GULO/GULOP* and *CLU* syntenic block (Fig. 4A). The *ADAM2* gene is frequently rearranged to precede the *EPHX2* gene in rodents. In other animal orders, *ADAM2* is not found at the chromosomal locus between *NUGGC* and *STMN4* which suggests that *ADAM2* is rearranged independently of the other syntenic blocks near *GULO*/*GULOP*. Lastly, within the Artiodactyl order, the *SCARA3* and *NUGGC* block is rearranged with a *APTX* and *BAG1* block. These data suggest frequent rearrangements around the *GULO*/*GULOP* locus which may impact cis-regulatory elements that act on *GULO*.

We further hypothesized that the inversions of *GULO* could be associated with the formation of *GULOP*. To test this hypothesis, the chromosomal and gene rearrangements belonging to each species were categorized as independent events as described in the methods section (Supplemental Fig. 1). We found that although most of the *GULOP* pseudogenes are located on the 5’ to 3’ strand, there is equal distribution of *GULO* on the 5’ to 3’ and the 3’ to 5’ strand. A p-value of 0.41 was calculated by Fischer’s exact test suggesting that the strand orientation of *GULO* does not associate with its pseudogenization.

**Fig. 5:**
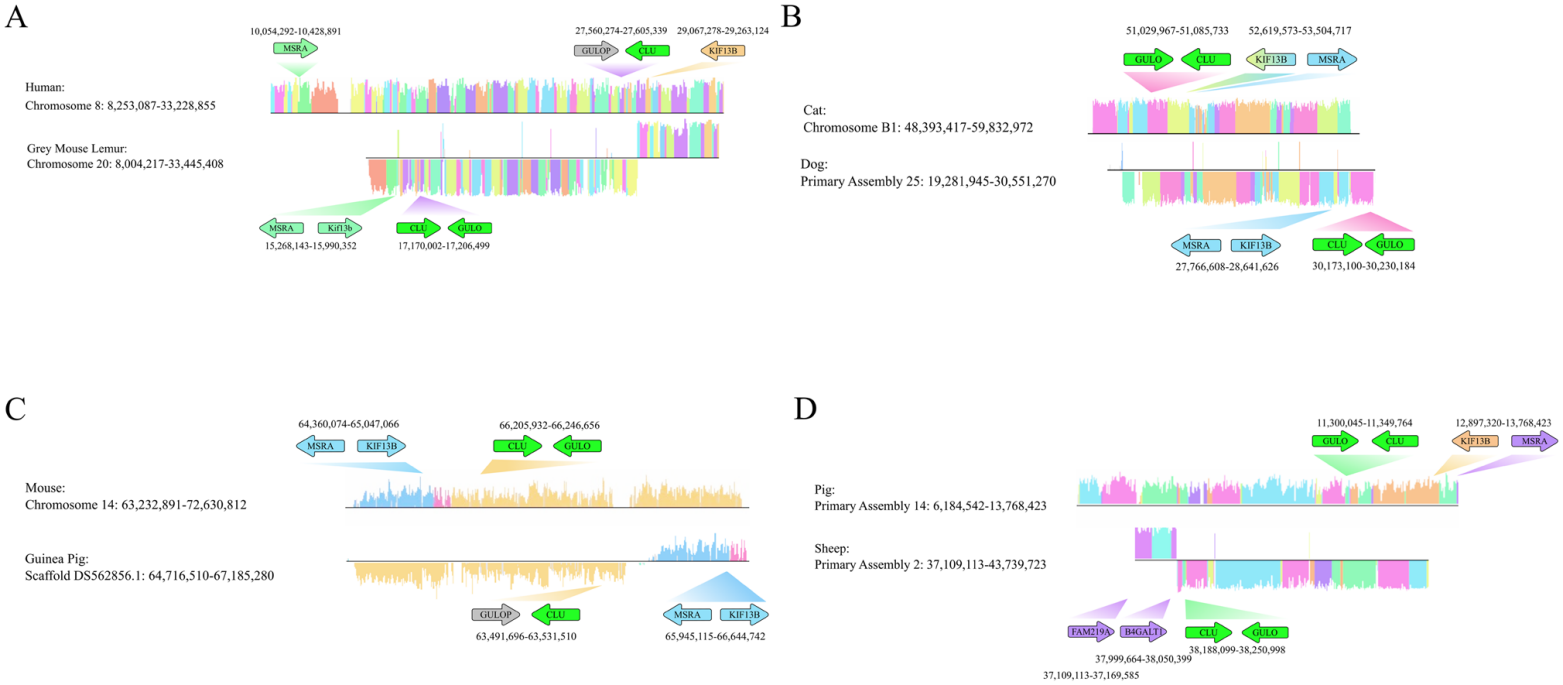
Mauve Whole Genome Alignment of The Syntenic Blocks Surrounding *GULO*/*GULOP*: The reference species is the first species shown for each figure panel. LCB orientation is depicted by the placement of gene blocks over or under the black center line. Blocks with inverse alignment are shown below the center line. LCB blocks refer to regions of the genome between species which are similar and potentially free of genomic rearrangement. LCB height is proportional to the conservation of the specific block when compared to the reference genome. **A** Comparison of human and grey mouse lemur chromosomes 8 and 20, respectively. **B** Comparison of cat and dog chromosomes B1 and 25, respectively. **C** Comparison of mouse and guinea pig chromosome 14 and DS562856, respectively. **D** Comparison of pig and sheep chromosomes 14 and 2, respectively

Next, we wanted to determine the breakpoints that resulted in the inversion. Whole chromosomes which contained the syntenic block of genes associated with *GULO* were analyzed by Mauve alignment. The domestic guinea pig genome is at a scaffold assembly level for this syntenic block and was the only scaffold assembly used. Using Mauve, the breakpoints between human and grey mouse lemur, cat and dog, mouse and guinea pig, and pig and sheep were identified (Supplemental Fig. 4). The breakpoints were found to be variable with the human containing the largest inverted segment of chromosomal DNA near the *GULOP* gene of ~ 16.5 million base pairs. The mouse and guinea pig were found to have the smallest inverted chromosome segment of ~ 7 million base pairs. From this analysis, it was found that the *KIF13B* and *MSRA* orthologues were syntenic and located about 1–2 million base pairs away from *GULO* and *CLU* (Fig. 5). In humans, the synteny between *KIF13B* and *MSRA* is disrupted with *MSRA* rearranged ~ 17 million base pairs away from *GULOP* (Fig. 5A). The gene orientation in cat and dog is reversed when compared between each other, but the overall synteny between *KIF13B* and *MSRA* is conserved (Fig. 5B). In the guinea pig scaffold, the *KIF13B* and *MSRA* block is rearranged to a slightly further position of ~ 2.5 million base pairs away from *GULOP* and on the opposite strand when compared to the mouse chromosome 14 segment (Fig. 5C). Lastly, the sheep *KIF13B* and *MSRA* gene segment is relocated ~ 37 million base pairs away (data not shown) from *GULO*, however an alternative set of genes is rearranged adjacent to the *GULO*-*CLU* syntenic block (Figs. 4 and 5D). This analysis may suggest that significant rearrangements of nearby gene blocks may occur more frequently in *GULO*-deficient animals.

## Discussion

*GULO*, which encodes the protein necessary to catalyze the final step in ascorbic acid synthesis, has undergone independent pseudogenization events in mammals. The *GULOP* sequence of haplorhini primates has been rigorously investigated in previous works (Lachapelle and Drouin 2011; Nishikimi and Yagi 1991; Ohta and Nishikimi 1999; Yang 2013). The work presented shows that a significant quantity of substitutions were conserved in the *GULOP* sequence of the haplorhini primates. This finding is supported by the long branch length of the haplorhini primates indicating a greater substitution per site in the suborder. In a study examining the pseudogenization of *UCP1* in placental mammals, a significantly greater substitution per site value was identified for the *UCP1* gene of placental mammals when compared to its paralogs *UCP2* and *UCP3* (Gaudry and Campbell 2017). It is suggested that the higher rate of substitutions in the *UCP1* gene was a result of relaxed purifying selection and enabled gain-of-function mutations for proton leakage. Though *GULO* is a unitary gene, diet can accommodate and replace endogenous ascorbic acid needs (Maeda et al. 2000). Therefore, the diet of haplorhini primates may have facilitated relaxed purifying selection or positive selection on *GULO* causing a significant accumulation of unique substitutions within the suborder. However, the substitution per site rate and total substitutions is reduced among species within the haplorhini suborder. Interestingly, miRNAs within processed pseudogenes of primates retain strong conservation and may conserve the underlying pseudogene the miRNA is derived from (Devor 2006). Yet, *GULOP* is an unprocessed pseudogene, which selection does not act on, so it is unclear how these former exons retain many conserved indels and substitutions. Taken together, these data suggest that the former *GULO* gene of haplorhini primates underwent a rapid substitution event at the beginning of the haplorhini/strepsirrhini split 70 MYA. The substitution per site rate has been reduced, and this may be a consequence of neutral evolution reducing a strong positive selection once experienced by the haplorhini *GULOP*. These data may provide insight towards identifying genes which may progress towards pseudogenization by having higher mutation rates when compared to orthologous genes in other species.

The investigation of SNVs between the human and neanderthal *GULOP* sequence revealed four SNVs. There is debate as to whether humans and Neanderthals are distinct species or subspecies. Despite these arguments, the human and Neanderthal populations are estimated to have split ~ 370,000 years ago (Noonan et al. 2006). Therefore, the neutral mutations that accumulated within *GULOP* occurred rapidly on an evolutionary time scale. The G96C SNV in the orthologous exon V of Neanderthals occurred at the last nucleotide position of the 3’ end which suggested that it could impact the exon donor recognition site. Exon/intron analysis of exon V reveals that this SNV does not impact the splice site. A caveat of this analysis is that the Neanderthal genome was sequenced from three bones acquired from Vindija samples (Green et al. 2010). Therefore, this sequencing represents a small sample when compared to the human genome sequencing project, and the SNVs in *GULOP* may not be representative of all Neanderthals.

When examining multiple *GULO*/*GULOP* sequences from placental mammals, the *GULO*/*GULOP* sequences are phylogenetically separated by order. This suggests overall conservation of the *GULO* gene. Indeed, previous findings have shown support for *GULO* conservation and for the evolution of *GULO* to conform to the current understanding of mammalian speciation (Yang 2013). Our findings extend upon previous work with the inclusion of the Brazilian guinea pig. The Brazilian guinea pig has an annotated *GULO* sequence in Ensembl™ version 107 which was used to identify the orthologous exon V region of the domestic guinea pig which was not originally identified (Nishikimi et al. 1992). Exon V of the Brazilian and domestic guinea pigs differ by a single indel which results in an in-frame or out-of-frame mutation, respectively. Both guinea pig species show a high rate of mutations within the rodent order, and without experimental testing, it is unknown if the Brazilian guinea pig can endogenously synthesize ascorbic acid. For these analyses, it was assumed that this was a functional gene transcript, despite the considerable genetic rearrangements which occurred within this *GULO* locus. Additionally, the pika was previously reported as having a functional *GULO* (Yang 2013). However, our findings suggest that the pika may not have a functional *GULO* gene. It would be interesting to examine guinea pig species and Lagomorphs for the ability to endogenously synthesize ascorbic acid as this may provide new information towards the evolution of *GULO* and the pseudogenes associated with it.

The *GULOP* of the chiroptera order which consists of bats has been studied in similar depth as the *GULOP* of haplorhini primates (Birney et al. 1976; Cui et al. 2011a, b; Drouin et al. 2011). However, two bat species, *Rousettus leschenaultia* and *Hipposideros armiger*, are thought to have independently regained a functional *GULO* (Drouin et al. 2011). In the present study, it is shown that there is significant gene conservation between the great roundleaf bat (*Hipposideros armiger*) and the greater horseshoe bat which contain *GULO* and *GULOP*, respectively. Interestingly, it has been shown that bats with *GULOP* can still express some Gulo protein, but only the Gulo protein from *Hipposideros armiger* was functional (Cui et al. 2011b). Cui and colleagues suggest that bat species may still produce L-gulonolactone oxidase, but the protein may be losing its function. The expression of a non-functional L-gulonolactone oxidase protein implies that the chiroptera promoter may be conserved. Taken together, the chiroptera order may have only recently lost *GULO*, and it is possible that drift has not altered much of the *GULOP* sequences or the promoter sequence. Functional promoters for human metallothionein pseudogenes have been identified despite these pseudogenes serving no known function similar to *GULOP* (Laukens et al. 2009). Therefore, regulatory elements of pseudogenes may remain intact, and this may facilitate pseudogenes regaining function as suggested by Drouin and colleagues (Drouin et al. 2011) or maintaining some degree of conservation as observed in this study.

While using BLAT analysis to find *GULOP* sequences in haplorhini primates, it was observed that the haplorhini *GULOP* was annotated in the 5’ to 3’ direction while the strepsirrhini *GULO* was annotated in the 3’ to 5’ direction. Additionally, a previously reported syntenic block for *GULO* suggested that *CLU* was syntenic with *GULO* (Yang 2013). Indeed, only the *CLU* gene is syntenic with *GULO*, and it was found to be inverted with *GULO* which suggested a larger chromosomal arrangement has occurred involving *GULO* and *CLU*. Chromosomal inversions play a significant role in evolution by preventing recombination during meiosis and facilitating speciation (Westram et al. 2022). A direct effect inversions may have on gene expression is by displacing enhancers or insulators from the respective gene target (Kleinjan and van Heyningen 1998). Therefore, a hypothesis was tested that this chromosomal inversion may be a causative factor for the pseudogenization of *GULO*. A chromosomal region containing *GULO* between the genes *STMN4* and *NUGGC* was examined. Several species with *GULOP* were consistently found to have a chromosomal rearrangement where *GULOP* ran 5’ to 3’. However, the *GULO* gene occurred on either strand with equal frequency. A chi-square test to determine if *GULO* pseudogenization was dependent upon *GULO* orientation did not achieve statistical significance.

Though the hypothesis was not supported, the chromosomal location around *GULO* has undergone several genomic rearrangements throughout evolution. Large chromosomal rearrangements are present within human evolution such as the 17q21.31 MAPT polymorphic inversion. In this polymorphism, the H1 configuration indicates the original orientation and the H2 configuration indicates the inverted configuration among human populations. However, it is the H2 configuration that is suggested to be the ancestral orientation and is similar to the syntenic region in chimpanzees (Zody et al. 2008). This implies that the H1 configuration is inverted relative to the ancestral state of this locus, and this finding is further supported by a greater number of polymorphic SNPs among the H1 orientation differing from the position matched H2 and ancestral alleles. These findings suggest that chromosomal inversions may increase nucleotide variability within populations and across species. It may be possible that the chromosomal arrangement of *GULOP* may influence some of the genetic variability observed within the haplorhini suborder and other species tested.

In addition to the inversion of the syntenic blocks around *GULO*, the *KIF13B* and *MSRA* genes are rearranged in human, guinea pig, and sheep genomes where *MSRA* or both *KIF13B* and *MSRA* are displaced from *GULO*. This frequent rearrangement may be partially responsible for the strand inversion the *GULO* syntenic block has undergone in evolution. Alternatively, the displacement of *MSRA* was of interest since it is expressed in liver and alleviates oxidative damage similar to *GULO* expression and indirectly related to the function of *GULO* (Harrison et al. 2010; Tabula Muris et al. 2018; Weissbach et al. 2005). There may be an enhancer region associated with the *MSRA* gene, which facilitates *GULO* expression, and its rearrangement may influence the expression of *GULO*. Animals like the sheep and cow have a dramatically altered genetic architecture near the *GULO* gene which displaces *MSRA*, but this region may provide a comparable cis-regulatory element to maintain *GULO* expression.

There are some weaknesses with the approach taken to test the impact of the chromosomal inversion on *GULO* pseudogenization. First, species where independent chromosomal inversions were selected by tracing the inversion taxonomically. For example, haplorhini primates show this chromosomal inversion suggesting that the inversion occurred during the haplorhini and strepsirrhini split. Therefore, this inversion event was only counted once to avoid redundant counting. Another weakness is regarding the accuracy of the chromosome assemblies used in this study. Species such as the greater horseshoe bat and Chinese rufous horseshoe bat belong to the same genus, but the chromosomal arrangements are different. These species were separated and counted as independent chromosomal rearrangement events. These species may have inaccuracies in the current assemblies provided for them resulting in the differences observed. Additionally, only a few full chromosome maps of animals tested for the Mauve alignment could be used in this study. This low sample number limited examining additional rearrangements along with the breakpoints surrounding the syntenic block *GULO* belongs to. As chromosome assemblies are improved with the rapid advancements in whole genome sequencing, a robust analysis can be performed to accurately examine the frequency of the chromosomal rearrangements and the occurrence of *GULOP*.

## Conclusion

*GULO* encodes the final protein for de novo vitamin C synthesis, and its loss is thought to occur as a neutral mutation in some organisms which can acquire sufficient dietary ascorbic acid. Our results to investigate nutritional evolutionary genetics has led to new findings, including previously undescribed SNVs in Neanderthals that have occurred in the *GULOP* sequences since the human and Neanderthal split 680,000 years ago (Pozzi et al. 2014). Neutral evolution, consisting of mutation and genetic drift, without selection has triggered rapid nucleotide changes between these sequences. In examining other extinct species such as *Denisovans* unique SNVs attributed to drift in GULOP may be identified. Additionally, there is a period of rapid nucleotide substitution between *GULO* sequences of strepsirrhini and haplorhini primates which is followed by a period of reduced substitution rates in the *GULOP* sequences. When examining the *GULO* evolution of other placental mammals, there are independent losses in guinea pigs, bats, and possibly pika. The Brazilian guinea pig has an open reading frame for *GULO* unlike the domestic guinea pig, but both sequences have undergone extensive rearrangements and substitutions. It is unknown if the Brazilian guinea pig retains a functional L-gulonolactone oxidase protein. Lastly, rearrangements of the *GULOP* locus attributed to chromosomal rearrangement may not be responsible for pseudogenization of *GULO*. Instead, the chromosomal rearrangement may have been conserved simultaneously with *GULOP* during haplorhini primate evolution.

### Supplementary Information

Below is the link to the electronic supplementary material.

Supplementary file1 (PDF 45 kb)

Supplementary file2 (PDF 58 kb)

Supplementary file3 (PDF 63 kb)

Supplementary file4 (DOCX 2167 kb)

## Data Availability

A list of common, abbreviated, and full genus species names is given in Supplemental File 1.

## Abbreviations

*GULO*: Gulonolactone oxidase
*GULOP*: Gulonolactone oxidase pseudogene
BLAT: Blast-like alignment tool
SNV: Single-nucleotide variant
MYA: Million years ago
MSA: Multiple sequence alignment
LCBs: Linear colinear blocks
