# Supplementary material for: Conservation of a Chromosome 8 Inversion and Exon Mutations Confirm Common Gulonolactone Oxidase Gene Evolution Among Primates, Including H. Neanderthalensis
Source: J Mol Evol. 2024 Apr 29;92(3):266–77. doi: 10.1007/s00239-024-10165-0 (PMC11169010; doi:10.1007/s00239-024-10165-0)
Supplement: Supplementary file 1 — Supplementary file1 (PDF 45 kb) [file 239_2024_10165_MOESM1_ESM.pdf]

Species Name	Common Name	Order	Sequence Acquired	Reciprocal BLAST Performed (Yes, Software, Subject)	Reciprocal BLAST Acquired	GULO Transcript Number	Transcript Name	Chromosome/scaffold	Genomic Legnth of GULO	GULO Functional	GULO Orientation	GULO/CLU Co-Occurrence	Clusterin Orientation
Microcebus murinus	Mouse Lemur	Primate	Ensembl 107	No	NA	1	ENSMICT00000053941.2	Chromosome 20	17,188,391-17,206,499	Yes	Reverse	Yes	Forward
Otolemur garnettii	Garnett's Galago	Primate	Ensembl 107	No	NA	1	ENSOGAT00000025787.1	Scaffold GL873593.1	3,059,738-3,080,850	Yes	Reverse	Yes	Forward
Propithecus coquereli	Coquerel's Sifaka	Primate	Ensembl 107	No	NA	1	ENSPCOT00000027847.1	Scaffold KQ028065.1	8,255,372-8,271,581	Yes	Forward	Yes	Reverse
Mus musculus	Mouse (C57Bl/6J)	Rodent	Ensembl 107	No	NA	1	ENSMUST00000059970.9	Chromosome 14	66,224,235-66,246,656	Yes	Reverse	Yes	Forward
Papio anubis	Olive Baboon	Primate	Ensembl 107	Yes, Ensembl 107, Human	Yes	0	NA	Chromosome 8	25,616,326-25,644,036	No	Forward	Yes	Reverse
Mandrillus leucophaeus	Drill	Primate	Ensembl 107	Yes, Ensembl 107, Human	Yes	0	NA	Scaffold KN974716.1	1,963,251-1,991,376	No	Forward	Yes	Reverse
Macaque mullata	Rhesus Macaque	Primate	Ensembl 107	Yes, Ensembl 107, Human	Yes	0	NA	Chromosome 8	27,825,501-27,852,698	No	Forward	Yes	Reverse
Pongo abelii	Sumatran Orangutan	Primate	Ensembl 107	Yes, Ensembl 107, Human	Yes	0	NA	Chromosome 8	26,519,211-26,548,307	No	Forward	Yes	Reverse
Pan troglodytes	Chimpanzee	Primate	Ensembl 107	Yes, Ensembl 107, Human	Yes	0	NA	Chromosome 8	27,014,181-27,043,083	No	Forward	Yes	Reverse
Pan paniscus	Bonobo	Primate	Ensembl 107	Yes, Ensembl 107, Human	Yes	0	NA	Chromosome 8	24,032,905-24,061,122	No	Forward	Yes	Reverse
Gorilla gorilla	Gorilla	Primate	Ensembl 107	Yes, Ensembl 107, Human	Yes	0	NA	Chromosome 8	26,656,774-26,685,728	No	Forward	Yes	Reverse
Homo sapiens	Human	Primate	Ensembl 107	No	NA	0	NA	Chromosome 8	27,560,272-27,560,378	No	Forward	Yes	Reverse
Homo neanderthalensis	Neandertal	Primate	Max Planck Institute for Evolutionary Athropology	Yes, Ensembl 107, Human	Yes	0	NA	Chromosome 8		No	Forward	Yes	Reverse
