# Supplementary material for: Conservation of a Chromosome 8 Inversion and Exon Mutations Confirm Common Gulonolactone Oxidase Gene Evolution Among Primates, Including H. Neanderthalensis
Source: J Mol Evol. 2024 Apr 29;92(3):266–77. doi: 10.1007/s00239-024-10165-0 (PMC11169010; doi:10.1007/s00239-024-10165-0)
Supplement: Supplementary file 3 — Supplementary file3 (PDF 63 kb) [file 239_2024_10165_MOESM3_ESM.pdf]

Species Name	Common Name	Order	Family	Sequence Acquired	Reciprocal BLAST Performed (Yes, Software, Subject)	Reciprocal BLAST Acquired	GULO Transcript Number	Transcript Name	Chromosome/scaffold	Genomic Legnth of GULO	GULO Functional	GULO Orientation	GULO/CLU Co-Occurrence	Clusterin Orientation	Notes
Bos taurus	Cow	Artiodactyla	Bovidae	Ensembl 107	No	NA	1		Primary Assembly 8	74,669,366-74,693,887	Yes	Forward	Yes	Reverse	
Ovis aries	Sheep (texel)	Artiodactyla	Bovidae	Ensembl 107	No	NA	1	ENSOART00000010494.1	Chromosome 2	37,997,301-38,020,786	Yes	Reverse	Yes	Forward	Two small genes between GULO and CLU
Delphinapterus leucas	Beluga Whale	Artiodactyla	Monodontidae	Ensembl 107	No	NA	1	ENSDLET00000001301.1	Primary assembly ML702014.1	10,876,042-10,905,071	Yes	Reverse	Yes	Forward	
Monodon monoceros	Narwhale	Artiodactyla	Monodontidae	Ensembl 107	No	NA	1	ENSMMNT00015026348.1	Primary assembly SIHG01006968.1	10,781,157-10,814,904	Yes	Reverse	Yes	Forward	
Phocoena sinus	Vaquita	Artiodactyla	Phocoenidae	Ensembl 107	No	NA	1	ENSPSNT00000018163.1	Primary assembly 6	104,876,722-104,906,483	Yes	Forward	Yes	Reverse	
Sus scrofa	Pig (Reference)	Artiodactyla	Suidae	Ensembl 107	No	NA	2	ENSSSCT00000010600.5	Primary assembly 14	11,300,045-11,336,139	Yes	Forward	Yes	Reverse	
Canis lupis familiaris	Dog (German Shephard)	Carnivore	Canidae	Ensembl 107	No	NA	2		Primary Assembly 25	30,175,908-30,207,796	Yes	Reverse	Yes	Forward	
Vulpes vulpes	Fox	Carnivore	Canidae	Ensembl 107	No	NA	1	ENSVVUT00000006063.1	Primary_assembly NBDQ01000068.1	4,633,631-4,665,823	Yes	Reverse	Yes	Forward	
Felis catus	Cat	Carnivore	Felidae	Ensembl 107	No	NA	1	ENSFCAT00000028823.4	Chromosome B1	51,029,967-51,062,748	Yes	Forward	Yes	Reverse	
Lynx canadensis	Canadian Lynx	Carnivore	Felidae	Ensembl 107	No	NA	1	ENSLCNT00005009362.1	Primary assembly B1	48,944,439-48,978,740	Yes	Forward	Yes	Reverse	
Zalophus californianus	California Sea Lion	Carnivore	Otariidae	Ensembl 107	No	NA	1	ENSZCAT00015003183.1	Primary assembly 2	153,632,000-153,663,132	Yes	Reverse	Yes	Forward	
Hipposideros armiger	Great Roundleaf Bat	Chiroptera	Hipposideridae	NCBI	No	NA	1		Unplaced Chromosome	363,791-417,651	Yes	Forward	Yes	Reverse	
Molossus molossus	Pallas's mastiff bat	Chiroptera	Molossidae	NCBI	No	NA	0	NA	GCF_014108415.1	82,558,666-82,590,582	No	Forward	Yes	Reverse	Only 10 exons are present
Pteropus alecto	black flying fox	Chiroptera	Pteropodidae	NCBI	No	NA	1	XM_015600680.1	GCF_000325575.1	5,113,284-5,138,269	No	Reverse	Yes	Forward	Encodes a protein of 286 AA
Pteropus giganteus	Indian flying fox	Chiroptera	Pteropodidae	NCBI	No	NA	1	XM_039875768.1	GCA_902729225.1	4,827,677-4,849,252	No	Reverse	Yes	Forward	Encodes a protein of 647 AA
Rousettus aegyptiacus	Egyptian rousette	Chiroptera	Pteropodidae	NCBI	No	NA	1	XM_016144624.2	NW_023416291.1	20,184,295-20,204,016	Yes	Forward	Yes	Reverse	12 SNPs occur between the Egyptian and Leschaulti's rousette species
Rhinolophus ferrumequinum	Greater Horseshoe Bat	Chiroptera	Rhinolophidae	Ensembl 107	No	NA	6		Primary Assembly 18	28,724,109-28,823,897	No	Forward	Yes	Reverse	Chose exons from hipposideros armiger BLAST against greater horseshoe bat which aligned to the transcript ID of ENSRFET00010021794.1. Did not include additional exons of this transcript.
Rhinolophus sinicus	Chinese rufous horseshoe bat	Chiroptera	Rhinolophidae	NCBI	No	NA	1	XM_019733088.1	GCF_001888835.1	6,599,375-6,630,440	No	Reverse	Yes	Forward	There are three different genes in the NCBI database. All are on the reverse strand. Selected the transcript adjacent to Clu
Oryctolagus cuniculus	Rabbit	Lagomorph	Leporidae	Ensembl 107	No	NA	2	ENSOCUT00000027132.3	Chromosome 2	42,085,515-42,110,122	Yes	Reverse	Yes	Forward	
Ochotona princeps	Pika	Lagomorph	Ochotonidae	Ensembl 107	Yes, NCBI, Oryctolagus cuniculus	Yes	0	NA	Gene Scaffold 936	320,944-339,721	Unlikely	Forward	Yes	Reverse	Chose sequences from reciprocal BLAST search to Oryctolagus cuniculus. Used NCBI fro reciprocal BLAST for relaxed alignment options
Monodelphis domestica	Opposum	Marsupial	Didelphidae	Ensembl 107	No	NA	1	ENSMODT00000020272.4	Primary_assembly 1	504,174,883-504,231,043	Yes	Reverse	Yes	Forward	
Microcebus murinus	Mouse Lemur	Primate	Cheirogaleidae	Ensembl 107	No	NA	1	ENSMICT00000053941.2	Chromosome 20	17,188,391-17,206,499	Yes	Reverse	Yes	Forward	
Otolemur garnettii	Garnett's Galago	Primate	Galagidae	Ensembl 107	No	NA	1	ENSOGAT00000025787.1	Scaffold GL873593.1	3,059,738-3,080,850	Yes	Reverse	Yes	Forward	
Homo sapiens	Human	Primate	Hominidae	Ensembl 107	No	NA	0	NA	Chromosome 8	27,560,272-27,560,378	No	Forward	Yes	Reverse	
Propithecus coquereli	Coquerel's Sifaka	Primate	Indriidae	Ensembl 107	No	NA	1	ENSPCOT00000027847.1	Scaffold KQ028065.1	8,255,372-8,271,581	Yes	Forward	Yes	Reverse	
Cavia aperea	Brazilian Guinea Pig	Rodent	Caviidae	Ensembl 107	No	NA	1	ENSCAPT00000007155.1	AVPZ01000112.1	63,458,181-63,472,619	Likely	Forward	Yes	Reverse	
Cavia porcellus	Domestic Guinea Pig	Rodent	Caviidae	Ensembl 107	Yes, Ensembl, Cavia aperea	Yes	0	NA	DS562856.1	63,491,696-63,506,078	No	Forward	Yes	Reverse	Recovered exons from Cavia aperea GULO transcript
Chinchilla lanigera	Chinchilla	Rodent	Chinchillidae	Ensembl 107	No	NA	2	ENSCLAT00000021123.1	Scaffold JH721863.1	50,132,178-50,154,632	Yes	Forward	Yes	Reverse	
Mesocricetus auratus	Golden Hamster	Rodent	Cricetidae	Ensembl 107	No	NA	1	ENSMAUT00000017046.1	Scaffold KB708180.1	2,434,830-2,454,258	Yes	Forward	Yes	Reverse	
Meriones unguiculatus	Mongolian Gerbil	Rodent	Muridae	Ensembl 107	No	NA	2	ENSMUGT00000031480.1	Primary_assembly NHTI01000032.1	1,397,210-1,415,754	Yes	Reverse	Yes	Forward	
Mus musculus	Mouse (C57Bl/6J)	Rodent	Muridae	Ensembl 107	No	NA	1	ENSMUST00000059970.9	Chromosome 14	66,224,235-66,246,656	Yes	Reverse	Yes	Forward	
Rattus norvegicus	Rat	Rodent	Muridae	Ensembl 107	No	NA	1	ENSRNOT00000022702.7	Primary assembly 15	40,205,665-40,227,874	Yes	Reverse	Yes	Forward	
Ictidomys tridecemlineatus	Striped Gopher	Rodent	Sciuridae	Ensembl 107	No	NA	1		Scaffold JH393485.1	470,978-492,441	Yes	Forward	Yes	Reverse	
