# Supplementary material for: Conservation of a Chromosome 8 Inversion and Exon Mutations Confirm Common Gulonolactone Oxidase Gene Evolution Among Primates, Including H. Neanderthalensis
Source: J Mol Evol. 2024 Apr 29;92(3):266–77. doi: 10.1007/s00239-024-10165-0 (PMC11169010; doi:10.1007/s00239-024-10165-0)
Supplement: Supplementary file 4 — Supplementary file4 (DOCX 2167 kb) [file 239_2024_10165_MOESM4_ESM.docx]

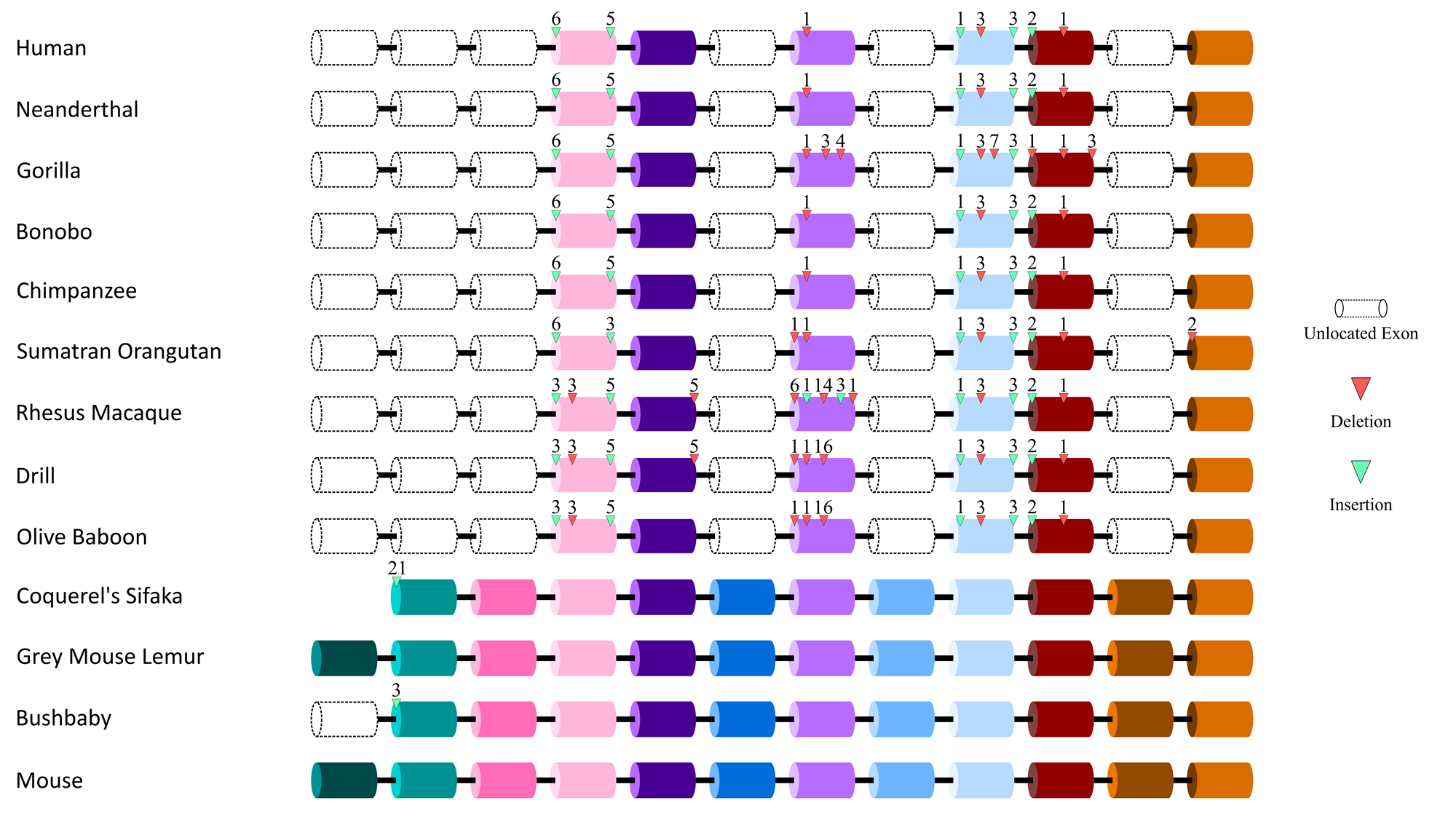


**Supplemental Figure 1: Exon schematic showing Indels (insertions: green, deletions: red).** The number of nucleotides inserted or deleted are shown above their respective triangles.


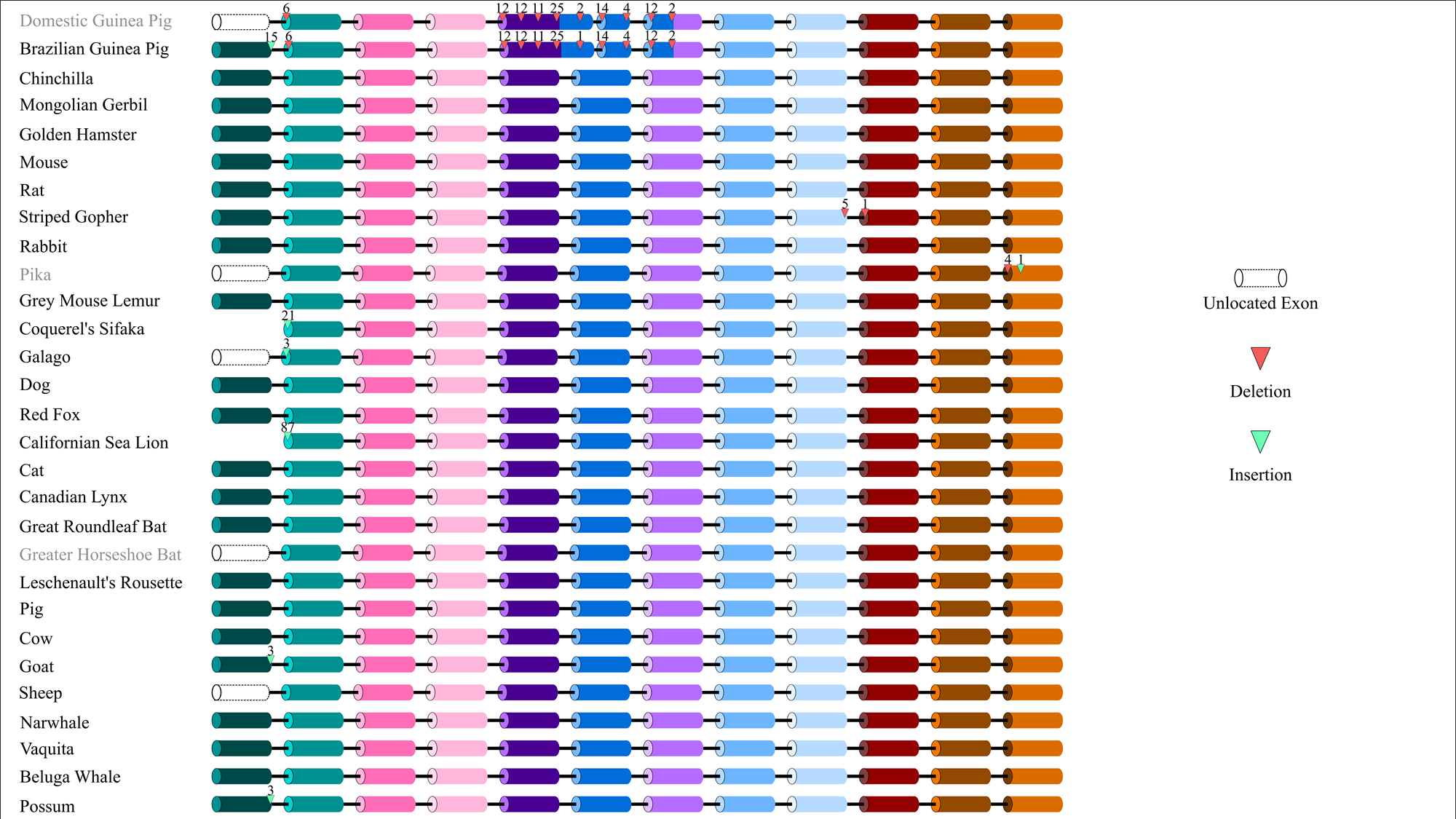


**Supplemental Figure 2: Exon schematic of well conserved *GULO* and *GULOP* sequences.** Indels are shown with red/green arrows for deletions and insertions, respectively. The number of nucleotides deleted or inserted are above their respective triangle. Species names colored in light grey do not have a functional or expressed *GULO* transcript and are considered pseudogenes.


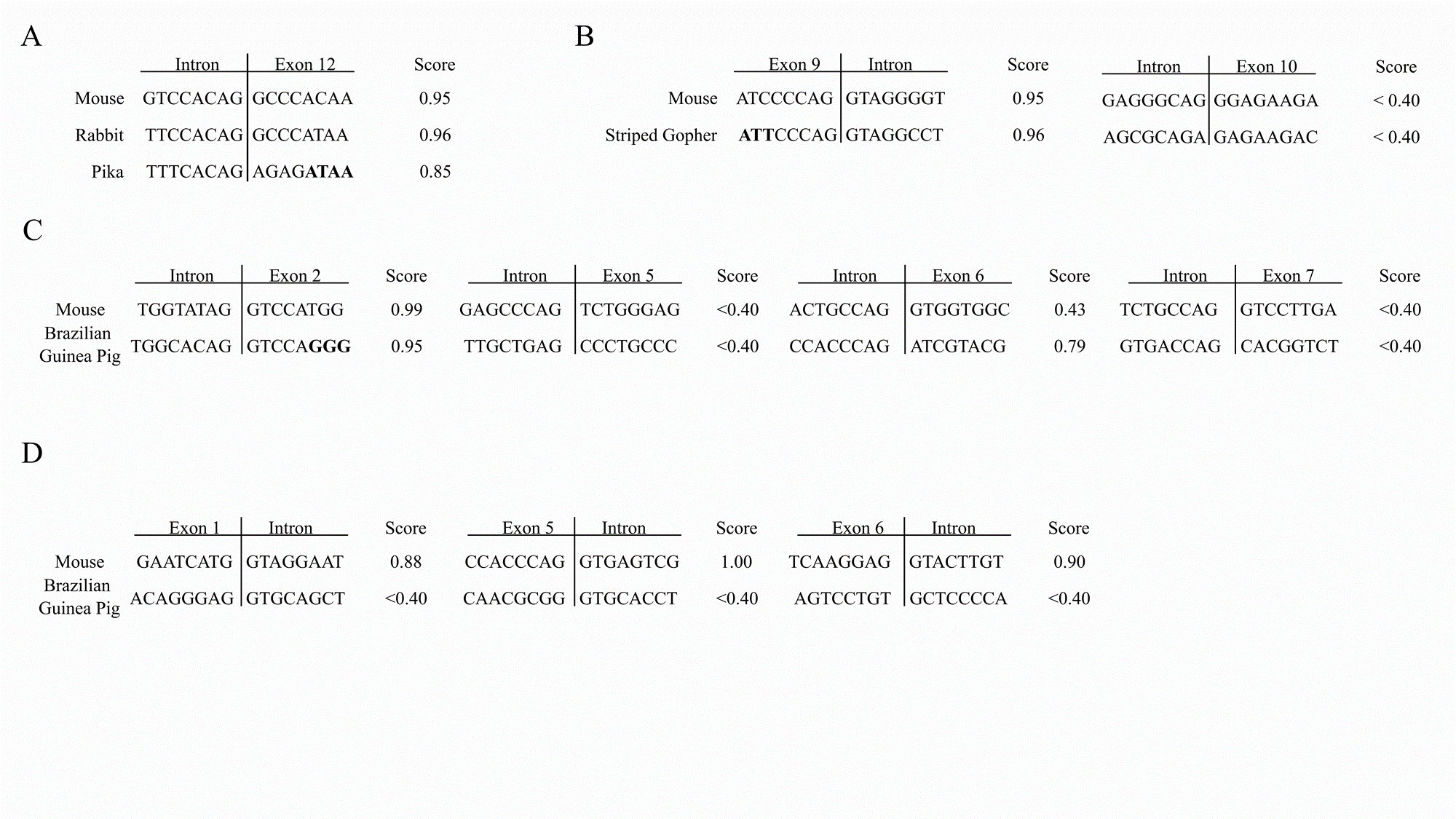


**Supplemental Figure 3: NNSplice 0.9 exon/intron analysis of sequences with exon loss at the immediate 5’ or 3’ ends.** NNSplice scores are reported on a scale of 0.00-1.00 with increasing values suggesting greater confidence in the predictions. Only sequences with scores of 0.40 or higher were reported in the analysis. (A) Analysis of the pika exon 12 4-bp deletion in *GULO*. (B) Analysis of the Exon 9 and 10 deletions in the striped gopher *GULO*. (C) Analysis of the exon acceptor splice sites in the Brazilian guinea pig. (D) Analysis of the exon donor splice sites in the Brazilian guinea pig. Sequences that are bolded show where the exon sequence starts or ends as acquired by reciprocal BLAST analysis.


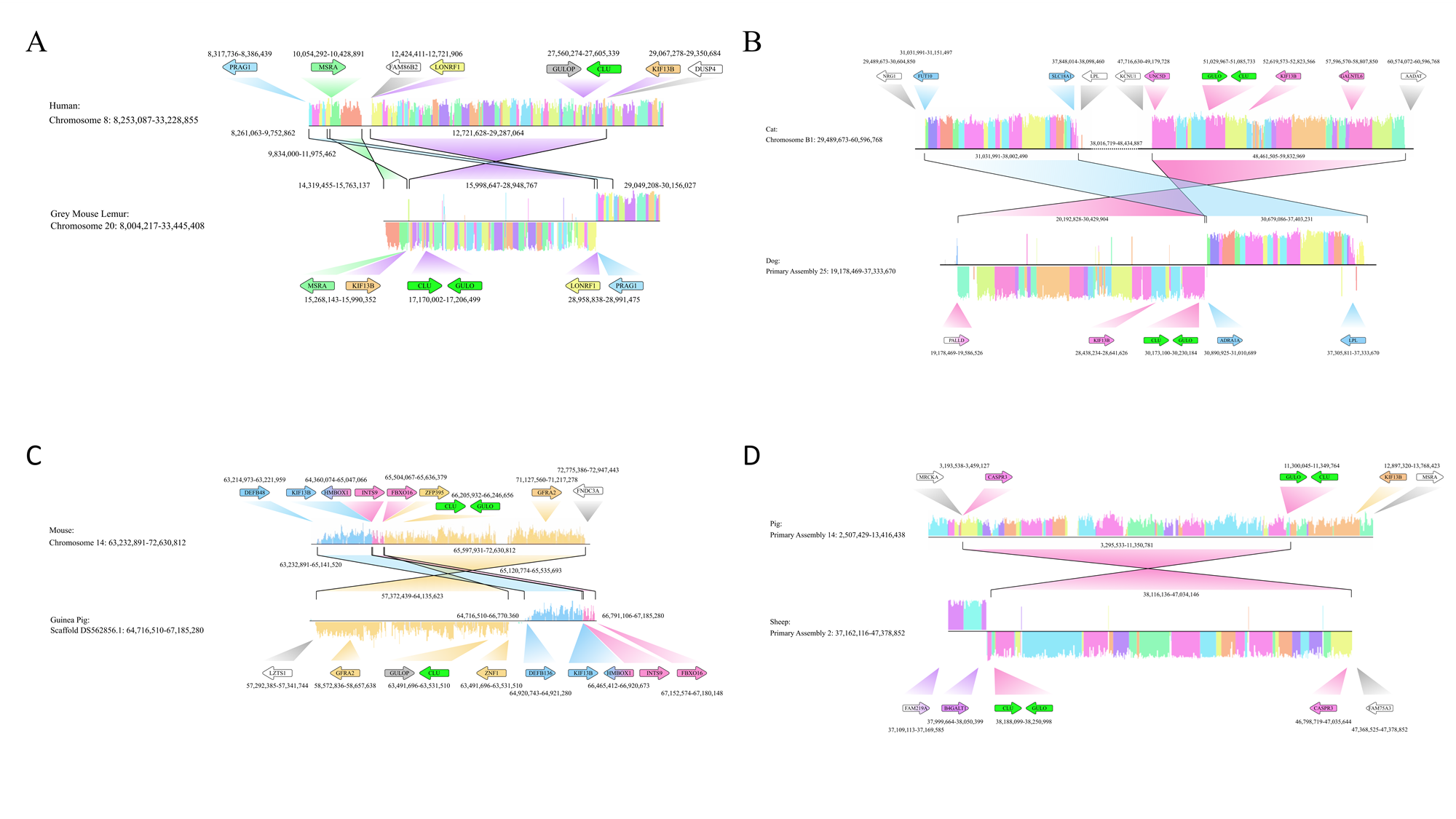


**Supplemental Figure 4: Mauve whole genome alignment of the GULO and CLU syntenic blocks on respective chromosomal regions.** Chromosome regions thought to encompass the core gene segment of the GULO and CLU syntenic block are annotated and shown reflected with the species they are compared with. Genes in white are the first genes occurring outside of regions of interest. The first and last gene of each block of interest is highlighted to create a map of breakpoints for each chromosome. A) Human chromosome 8 is the reference chromosome and compared with the grey mouse lemur chromosome 20. B) The cat chromosome B1 is the reference chromosome and compared to the primary assembly of chromosome 25 of the dog reference breed. C) The C57Bl/6J mouse chromosome 14 is used as the reference chromosome and compared with the guinea pig scaffold DS562856.1. D) The primary assembly for chromosome 14 of the reference pig genome is used as a reference and compared with the sheep primary assembly 2.
